# Prebiotic Chemistry that Could Not *Not* Have Happened

**DOI:** 10.3390/life9040084

**Published:** 2019-11-14

**Authors:** Steven A. Benner, Hyo-Joong Kim, Elisa Biondi

**Affiliations:** 1 Foundation for Applied Molecular Evolution, 13709 Progress Blvd. Box 7, Alachua, FL 32615, USA; 2 Firebird Biomolecular Sciences LLC, 13709 Progress Blvd. Box 17, Alachua, FL 32615, USA

**Keywords:** prebiotic chemistry, RNA formation, organic minerals, Hadean, meteorite impact

## Abstract

We present a direct route by which RNA might have emerged in the Hadean from a fayalite–magnetite mantle, volcanic SO_2_ gas, and well-accepted processes that must have created substantial amounts of HCHO and catalytic amounts of glycolaldehyde in the Hadean atmosphere. In chemistry that could not *not* have happened, these would have generated stable bisulfite addition products that must have rained to the surface, where they unavoidably would have slowly released reactive species that generated higher carbohydrates. The formation of higher carbohydrates is self-limited by bisulfite formation, while borate minerals may have controlled aldol reactions that occurred on any semi-arid surface to capture that precipitation. All of these processes have well-studied laboratory correlates. Further, any semi-arid land with phosphate should have had phosphate anhydrides that, with NH_3_, gave carbohydrate derivatives that directly react with nucleobases to form the canonical nucleosides. These are phosphorylated by magnesium borophosphate minerals (e.g., lüneburgite) and/or trimetaphosphate-borate with Ni^2+^ catalysis to give nucleoside 5′-diphosphates, which oligomerize to RNA via a variety of mechanisms. The reduced precursors that are required to form the nucleobases came, in this path-hypothesis, from one or more mid-sized (10^23^–10^20^ kg) impactors that almost certainly arrived after the Moon-forming event. Their iron metal content almost certainly generated ammonia, nucleobase precursors, and other reduced species in the Hadean atmosphere after it transiently placed the atmosphere out of redox equilibrium with the mantle. In addition to the inevitability of steps in this path-hypothesis on a Hadean Earth if it had semi-arid land, these processes may also have occurred on Mars. Adapted from a lecture by the Corresponding Author at the All-Russia Science Festival at the Lomonosov Moscow State University on 12 October 2019, and is an outcome of a three year project supported by the John Templeton Foundation and the NASA Astrobiology program. Dedicated to David Deamer, on the occasion of his 80th Birthday.

## 1. Introduction

The ribonucleic acid (RNA) catalysts and cofactors found widely in Earth’s biosphere today support the view that an early episode of life on Earth used RNA for both genetics and catalysis [1,2,3]. This episode has been called an “RNA World” [4]. Analyses of the genomic sequences of modern organisms have allowed models for its metabolism to be adumbrated [5].

These models, in turn, led to the strong “RNA First” hypothesis for the origin of Darwinian evolution on Earth [6]. Darwinian evolution is thought to be the only mechanism for matter to self-organize to produce the behaviors that we value in life. It requires (at least) a molecular system that supports a line of descent via replication with non-prospective errors, errors that are themselves replicable. Those errors are naturally selected if adaptive, are removed if disadvantageous, and drift randomly if neither.

The nature of the gases emerging from Hadean volcanoes offers a constraint on the prebiotic chemistry that might have occurred in the Hadean atmosphere to make the first RNA molecules. Efforts to define those gases have focused on the mineralogy of the early Earth. From isotope data, it seems that Earth accreted primarily from enstatites that dominated the pre-solar nebula ~150 million km from its center [7]. This oxygen-poor material is in redox equilibrium with CO and CH_4_ rather than CO_2_, with NH_3_ rather than N_2_, with H_2_ rather than H_2_O, and with H_2_S rather than SO_2_. Thus, gases expected to emerge from a planet made of enstatites are similar to those used by Stanley Miller in his experiments that formed aldehydes, cyanide, and amino acids by electrical discharge [8].

Unfortunately for models based on Miller experiments, the mantle (and its emerging gases) did not remain reducing for long. As freshly accreted at 4.53 Ga and, again, after the Moon-forming event at 4.51 Ga [9,10,11,12], Earth was largely molten. Its molten metallic iron almost certainly fell to the core, where it is today [13]. This core “closure” was rapid, as shown by the siderophilic ^182^W that remains in the mantle following decay (with an 8.9 My half-life) of the lithophilic ^182^Hf [14,15]. Thus, within (at most) a few tens of millions of years, the Earth’s mantle became much more oxidizing, approaching the fayalite–magnetite–quartz (FMQ) level [16]. This means that the gases emerging from Hadean volcanoes comprised primarily CO_2_ rather than CO or CH_4_, primarily N_2_ rather than NH_3_, primarily H_2_O rather than H_2_, and primarily SO_2_ rather than H_2_S. This is confirmed by studies of the ratios of Ce^4+^/Ce^3+^ in Hadean zircons, which show that by ~4.35 Ga, the Earth’s mantle had a fayalite–magnetite–quartz (*f*_O2_ = FMQ − 0.5 ± 2.3) oxygen fugacity [17].

This “redox neutral” atmosphere is quite unproductive in Miller-type syntheses. For example, the production of HCN drops to zero as the H_2_:CO_2_ ratio drops to zero [18]. To make useful amounts of larger molecular precursors of RNA bases, such as H_2_NCN (cyanamide), HCCCN (cyanoacetylene), NCCN (cyanogen), and species that emerge via hydrolysis of these (e.g., NH_3_, urea, formamide), even more reducing atmospheres appear necessary.

Nevertheless, these reduced molecules have long been invoked in prebiotic syntheses [19], and still remain staples of path-hypotheses for the synthesis of RNA precursors. For example, recent thoughts from the Simons Collaboration on the origin of carbohydrates propose a multi-step Fischer–Kiliani synthesis that requires stoichiometric HCN at each step [20,21]. Since HCN (and ferrocyanide, under many conditions [22,23]) hydrolyzes with measured rate constants of 10^−5^ to 10^−1^ day^−1^ (at 60 °C from pH 4 to 9) [24], accumulation of HCN over geological time does not mitigate a poorly productive atmosphere, a fact that has caused some to consider non-atmospheric sources of HCN [25,26].

Unfortunately, subsequent processes in the Hadean occurring over geological time likely made its atmospheres still more oxidizing, and still less able to support Miller-type syntheses. Thus, in the mantle, Fe^2+^ disproportionates to give Fe^3+^ and Fe^0^ [27]. The first remains in an increasingly more oxidizing mantle as the second falls towards the core. In the atmosphere, photochemical processes generate hydrogen radicals; these combine to produce H_2_ molecules, which evaporate into space, increasing the oxidation state of the atmosphere as well [28].

Some prebiotically useful compounds are nevertheless made in redox-neutral atmospheres. For example, formaldehyde (HCHO) is formed from CO_2_ and H_2_O via UV light or electrical discharge; the amount of its production is largely independent of the redox potential of the gas mixture [29]. Further, unlike with HCN, HCHO has no hydrolytic path to destruction. Aqueous HCHO is stable against self-reaction, if the pH is not so high as to allow Cannizzaro reactions.

In contrast, atmospheric glycolaldehyde production [30] depends strongly on the amount of admixed reducing gases [29]. Even with these, glycolaldehyde formation remains inefficient. For example; with a CH_4_:CO_2_ ratio of 0.02, the glycolaldehyde:HCHO ratio is only ~10^−6^. This may be sufficient for glycolaldehyde to play a catalytic role in prebiotic chemistry [6,31]. However, as Harman et al. note [29], atmospheric glycolaldehyde is not easily made in amounts sufficient to be a stoichiometric participant in the formation of RNA building blocks, as required by some models [32]. Adding to its troubles, glycolaldehyde easily enolizes to give a species that self-reacts in aldol reactions. This strongly constrains its lifetime in aqueous media. Thus, at pH 10.5 where ferrocyanide is stable, the half-life for enolization and subsequent reaction of glycolaldehyde is in the order of minutes [33].

## 2. Unavoidable Prebiotic Chemistry of Carbonyl C = O Groups

**Figure 1:**
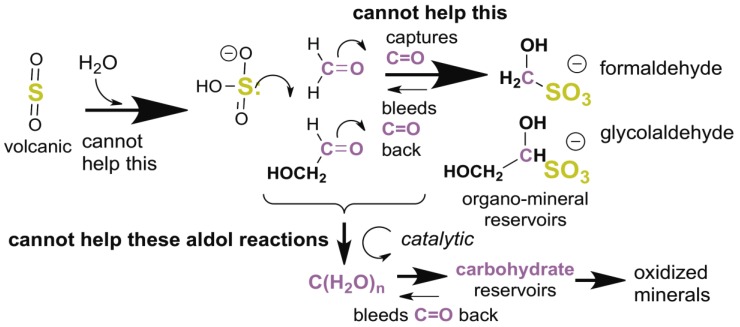
Volcanic sulfur dioxide (SO_2_) becomes sulfurous acid (H_2_SO_3_) in water aerosol particles. With any alkali, this forms the bisulfite anion (HSO_3_^−^). This, in turn, reacts with carbonyl C=O groups to give sulfonate “bisulfite addition products”. The sulfonates are quite stable to self-reaction and other degradative paths. Their only reaction at modest temperatures is a reversible dissociation to give back carbonyl compounds. Thus, sulfonates slowly bleed reactive C=O species into aqueous mixtures [35].

However, these facts about the Hadean suggest a different, and nearly unavoidable, fate for much of the atmospherically generated HCHO and other C = O carbonyl compounds: they reversibly react with volcanic SO_2_ to form sulfonates having the general formula R–CH(OH)SO_3_ [34] (Figure 1). This reaction occurs in atmospheric aerosols above Earth today, with HCHO and SO_2_ being products of human activities (e.g., coal burning). In aqueous media containing at least micromolar concentrations of these, the reaction would have been unavoidable in the Hadean as well.

This “privileged” or “bespoke” (that is, hard to avoid) chemistry is well studied in both modern Earth atmospheres and in laboratories [36,37,38]. Thus, the equilibrium constant for sulfonate dissociation ranges from 2 × 10^−4^ M (pH 10 [37]) to 1 × 10^−7^ M (pH 4 [38]) ([SO_3_^−^]_tot_·[HCHO]_tot_/[HOCH_2_SO_3_^−^]). In the absence of a buffer, the pH of water droplets absorbing SO_2_ is ~4. This is key to models describing the fate of prebiotically formed carbonyl compounds in atmospheres containing volcanic SO_2_.

With a rate of volcanic delivery of SO_2_ to the Hadean atmosphere comparable to the rate of generation of Hadean atmospheric carbonyl compounds much of the atmosphere-generated HCHO would have precipitated to Earth as its bisulfite addition products. These products lack C=O groups, and thus are stable with respect to many reactions, including much longer wave photochemistry. Their only mode of decomposition in water is the reverse reaction, to bleed reactive HCHO into the aquifer.

**Figure 2:**
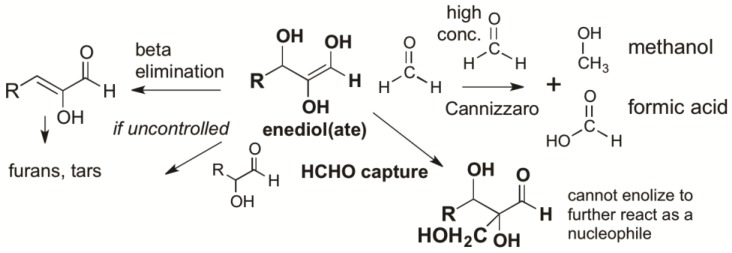
Formaldehyde (HCHO) captures enediol(ate)s formed from C=O carbonyl compounds before they react to give complex mixtures of unproductive species. At high concentrations, HCHO disproportionates in a bimolecular Cannizzaro reaction to give unproductive methanol and formate. However, these high concentrations are not possible in the presence of bisulfite, as the organic sulfonate mineral bleeds only small amounts of HCHO into a prebiotic reaction mixture. Glycolaldehyde is likewise bled into the mixture, but only in catalytic amounts, as its atmospheric formation is far less efficient. Glycolaldehyde cannot accumulate in aqueous alkaline environments because it enolizes under these conditions, a reaction that occurs faster than a Cannizzaro reaction.

Here, the high reactivity and relative abundance of atmosphere-formed HCHO plays a role in managing the intrinsic instability of carbonyl compounds. Even at low concentrations (10 mM) leaked from its bisulfite addition product, HCHO traps enolates from C=O compounds, preventing beta eliminations, hydride shifts [39], self-reactions, and other processes that give “tars” (Figure 2) [31]. HCHO at pH ~10.5 even prevents the protonation of enolates [33]. Thus, in a quantitative study, Kim et al. [31] showed that low concentrations of HCHO would react as the preferred electrophile with any enol formed by any aldose or ketose. Only after HCHO was consumed does the “yellowing” characteristic of tar formation begin [40].

This is true in glycolaldehyde, which was also undoubtedly produced in the Hadean atmosphere, but only in small (or very small) amounts. Glycolaldehyde is useful in the prebiotic formation of ribose and other carbohydrates, as it can act catalytically to fix HCHO [6,31]. However, pools of glycolaldehyde cannot form at elevated pH, as it rapidly enolizes to an enediol that reacts with glycolaldehyde to form tetroses and, then, higher products [31]. As noted above, the half-life for the enolization in this process is in the order of minutes at pH 10.5 [33].

**Figure 3:**
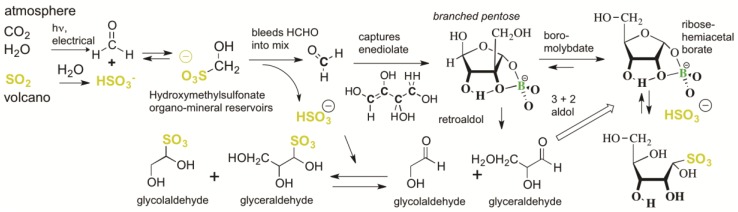
Unavoidable reactions involving volcanic SO_2_, HCHO, and other lower carbohydrates in the presence of borate. With each C–C bond formed from a C=O species that comes from a sulfonate, a bisulfite molecule is released. An increasing bisulfite concentration causes the process to be self-limiting; higher and higher concentrations of bisulfite increase the equilibrium levels of unreactive sulfonates. Borate forms a cyclic adduct with the indicated branched pentose, which can undergo a retroaldol reaction to give glycolaldehyde and glyceraldehyde. These can either combine directly to give ribose [42], or can enolize to fix more HCHO in a catalytic cycle [6]. Reaction of bisulfite with ribose competes with ring closure. Thus, bisulfite addition with ribose is less than with HCHO, glycolaldehyde, and glyceraldehyde. The same is the case with ketoses such as xylulose and ribulose. The stereochemistry shown is entirely arbitrary; in the absence of processes for stereo-control, these compounds are made as racemates.

However, if HCHO is present, the enol of glycolaldehyde is unavoidably captured by HCHO to form glyceraldehyde, a three-carbon carbohydrate. Again, if HCHO is present, the enol of glyceraldehyde can escape tar formation by reacting with HCHO to form a four-carbon species (erythulose, Figure 3). If borate is present, its binding to two adjacent hydroxyl groups (1,2-diols) in various of these species further controls downstream reactions, diminishing “tar” formation [31]. Alternatively, all downstream C=O species can react with SO_2_ to form their bisulfite addition products, which are stable (except with respect to the reverse reaction).

Usefully, this process is self-quenching. Every time a new carbon–carbon bond is formed via an aldol addition reaction of species arising from the dissociation of a sulfonate precursor, a dissolved SO_2_ molecule (as bisulfite) is released without a C=O partner. Thus, as the aldol reactions proceed in a closed system, the unstable C=O species must encounter higher and higher concentrations of bisulfite, preventing the formation of tar. This is illustrated in Figure 3. These reactions are also pH-dependent. An aquifer having access to basalts would raise the pH to 7–9.5, depending on their exact composition [41]. These are conditions where the formation of higher carbohydrates from HCHO and trace glycolaldehyde would have been difficult to avoid.

**Figure 4:**
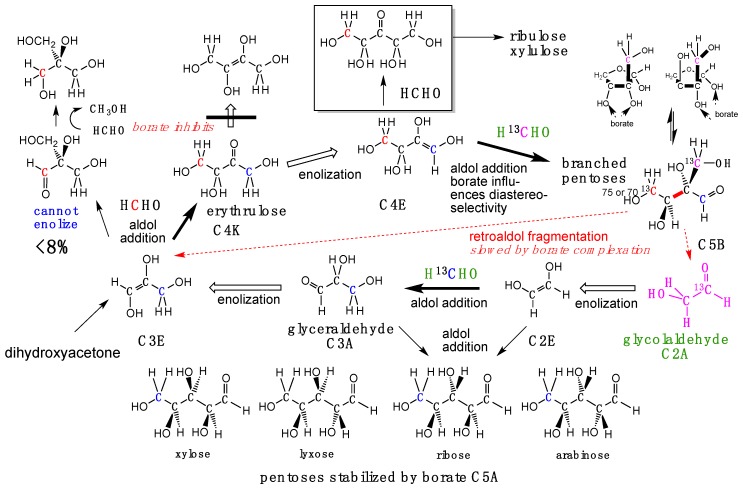
Prebiotic aldol reactions that are unavoidable in the presence of borate, which guides enolization of four-carbon carbohydrates and directs the regiochemistry of attack of HCHO on their enols.

Having special impact on these processes are borate minerals such as kernite, ulexite, and colemanite. These are relatively soluble in water, releasing borate that complexes with 1,2-diols of the chemically evolving carbohydrates (Figure 3). This guides the outcome of the reaction towards, in particular, branched pentoses [31] (Figure 4).

Branched pentoses form cyclic hemiacetals that themselves are good ligands for borate. They are also metastable intermediates. As they cannot enolize, their only mode of reaction is via a retroaldol reaction to generate one molecule each of glyceraldehyde and glycolaldehyde (Figure 4). These two can, of course, react directly with each other to form ribose [41], or can serve as the starting points for more HCHO fixation, making the cycle catalytic [6,31].

This overall process in the presence of bisulfite was studied experimentally by Kawai et al. [38], who examined the reaction manifold using ^13^C-labeling. Here, sodium carbonate–bicarbonate buffers at pH 10.5 were used to represent aquifers beneath a CO_2_ atmosphere and above serpentinizing basalts, with the pH modestly higher than that expected in Hadean atmospheres to make the reactions proceed at an easily studied rate. The bisulfite adduct of HCHO (hydroxymethylsulfonate, HMS), by slowly dissociating to give free HCHO, captures the enolate of three-carbon carbohydrates before they can self-react to give (for example) 4-hydroxymethyl-1,3,4,5-tetrahydroxy pentane-2-one, on the way to tar.

Of course, the high water solubility of the sodium salt of HMS (~1 g/mL at 25 °C) means that it could accumulate as a solid only in an arid desert environment. For example, the solubility of barium bis(hydroxymethanesulfonate) (BaC_2_H_6_S_2_O_8_) in water at 25 °C is 386 g/L, about the same as NaCl [38]. These “organic minerals” are not known on Earth today, as its biosphere would rapidly consume them if they were formed.

As noted above, volcanic SO_2_ can form bisulfite addition products with glycolaldehyde, glyceraldehyde, dihydroxyacetone, erythrulose, and even ribose [38]. The addition of bisulfite to glycolaldehyde and glyceraldehyde, while also reversible, goes largely to completion with a dissociation constant of less than ~10^−5^ M. The bisulfite addition products of dihydroxyacetone (where the C=O unit is in the form of a ketone) and ribose are thermodynamically less stable. For the first, this is explained using a steric model. Just as the hydrate of dihydroxyacetone is less stable that the hydrate of glyceraldehyde, so is its bisulfite addition product. The relatively little bisulfite addition product formed by ribose is understood by its need to compete with its cyclic forms. In the presence of borate, this competition is even less favorable, since the cyclic forms are especially strong ligands for borate.

To complete the catalytic cycle [6], the branched pentoses undergo slow retroaldol reactions in the presence of borate, and rapid retroaldol reaction in the absence of borate (Figure 3). An aquifer would have had differential access to an effectively unlimited amount of HCHO and its derived products coming from the atmosphere, and borate coming from the lithosphere. As long as borate is present in excess over branched pentose, the branched pentose is stabilized. However, as HCHO fixation proceeds, the concentrations of branched pentose molecules may eventually surpass the concentration of available borate; these will undergo the retroaldol reaction.

**Figure 5:**
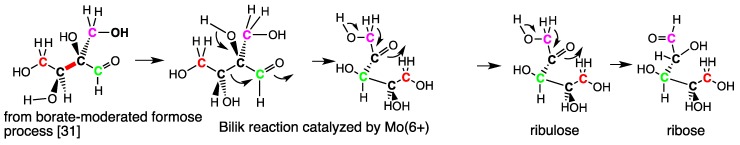
The reversible conversion of branched pentoses at pH 6 (25 °C) catalyzed by Mo^6+^ gives an alternative path for the creation of linear pentoses and pentuloses [44]. Again, stereochemistry is entirely arbitrary. However, the rearrangement is itself stereospecific.

**Figure 6:**
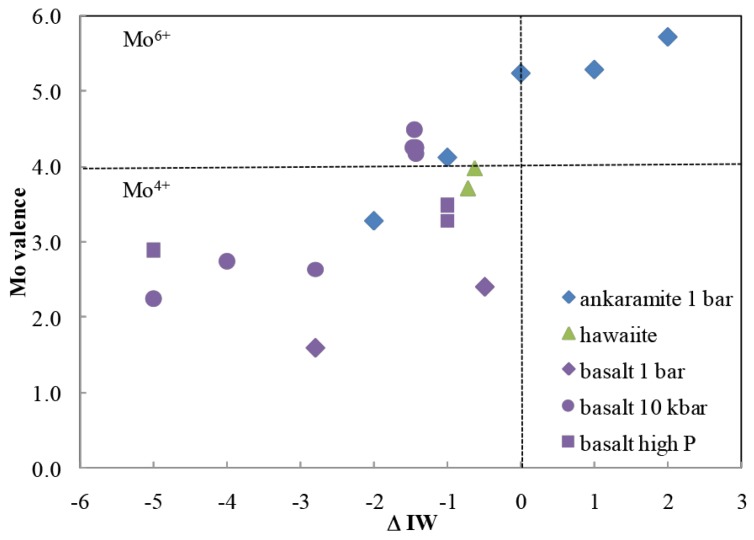
Molybendum in its +6 oxidation state can be obtained from melts having a redox potential greater than the iron–wüstite oxygen fugacity, which is several of orders of magnitude below the fayalite–magnetite–quartz (FMQ) fugacity likely present in the Hadean mantle. From Reference [45].

The branched pentose is, of course, an isomer of ribose (or arabinose, ribulose, or any of a number of other ketopentoses and aldopentoses). Via the retroaldol reaction, a linear pentose (e.g., ribose) can be formed without any special catalyst [41]. However, branched pentoses can also be converted to linear species by molybdenum at the +6 redox state [43] (Figure 5). Here, the pH is 6–7, temperature is low, only minutes are required, and no tar is formed. Such processes are uncommon in carbohydrate chemistry. Again, Mo^6+^ can be the dominant oxidation state of molybdenum in FMQ melts (Figure 6), depending on their detailed compositions. Thus, the Hadean mantle likely had Mo^+6^, likely in only catalytic amounts. Thus, it is not clear the extent to which a Mo^6+^-catalyzed process competed with a retroaldol process to create linear pentoses from branched pentoses.

**Figure 7:**
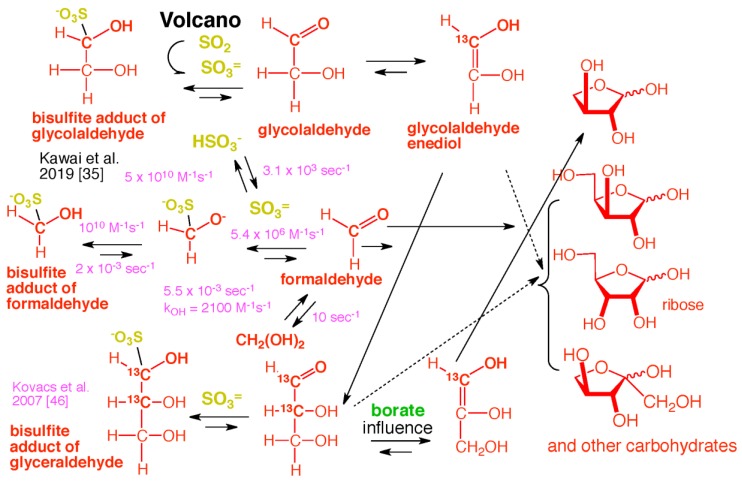
Overall “bespoke” chemical evolution of carbohydrate species that is hard to avoid in a Hadean environment that had semi-arid subaerial surface to capture precipitating bisulfite addition products formed from volcanic SO_2_ and atmospherically generated carbonyl compounds [35,46]. Again, stereochemistry is entirely arbitrary. Some key rate constants are shown.

Figure 7 presents an overall picture for the chemical evolution of carbohydrate species in this Hadean environment. Again, much of this chemistry could not *not* have happened in that environment.

## 3. Unavoidable Prebiotic Chemistry of Carbohydrate Phosphates

The fate of carbonyl C=O compounds, either stabilized by borate or as bisulfite addition products, that accumulated on any semi-arid sub-aerial surface of the Hadean Earth then becomes tied to the status of phosphorus there. Phosphoric acid itself, upon drying, forms polyphosphates and cyclic phosphates. Cyclic trimetaphosphate has long been interesting to prebiotic chemists, who see this as a ready precursor of nucleoside triphosphates.

**Figure 8:**
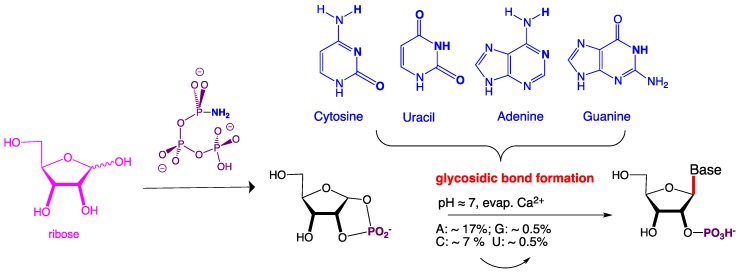
The reaction of amidotriphosphate with ribose gives a 1,2-cyclic phosphate. This reacts in the presence of Ca^2+^ upon evaporation in urea to give the canonical nucleosides.

The direct triphosphorylation of nucleosides by cyclic trimetaphosphate has proven to be elusive [47]. However, two decades ago, Krishnamurthy et al. showed that in the presence of ammonia, cyclic trimetaphosphate forms an acyclic amide [48] (Figure 8). The P–NH_2_ group (unlike the C=O–NH_2_ group) forms an imine with C=O groups of carbohydrates. If these have an adjacent hydroxyl group, that group is phosphorylated. This forms a direct route to 1,2-cyclic species (Figure 7). Other phosphoramides have analogous reactivity [48].

The cyclic phosphate of ribose (and also of other carbohydrates, such as threose, a four-carbon aldotetrose) proves to have interesting reactivity. In particular, upon evaporation in the presence of Ca^2+^, the cyclic phosphate directly reacts with many nucleobases to form nucleoside-2′-phosphates via the formation of the glycosidic bond [49]. This bond too has been regarded as problematic, dating all of the way back to Orgel, and is classified as an “Orgel hard” problem. Indeed, several multistep processes have been constructed specifically to manage the presumption that this bond cannot be formed directly, especially from pyrimidines [43], or regioselectively [50].

Again, such direct glycosyl bond-forming reactions could hardly not have happened on the Hadean Earth to any carbohydrates (including ribose and threose) that found themselves on a semi-arid surface in the presence of polyphosphates (especially cyclic trimetaphosphate) and NH_3_. Such surfaces almost certainly contained Ca^2+^ and would have been modestly hot, as in today’s Death Valley.

**Figure 9:**
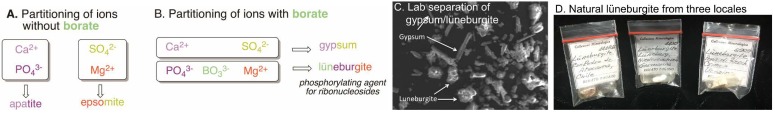
(**A**) Absent borate. Ca^2+^ and PO_4_^3–^ segregate to give apatite, leaving behind Mg^2+^ and SO_4_^2-^ to form epsomite, (**B**), in the presence of BO_3_^3–^, Mg^2+^, BO_3_^3–^ and PO_4_^3–^ segregate to give lüneburgite, leaving behind Ca^2+^ and SO_4_^2-^ to form gypsum. (**C**) The segregation of gypsum and lüneburgite is seen in laboratory experiments. (**D**) Lüneburgite samples from three locations on the modern Earth, Chile, Crimea, and (from the type-locality) Germany. Samples are courtesy of Renato Pagano and Robert Lavinsky (The Arkenstone, Richardson TX, https://www.irocks.com/), illustrating the importance of commercial and amateur “rock hounds” in the development of this science.

The status of calcium, phosphate, borate, and other cations (in particular Mg^2+^) on a semi-arid surface on the Hadean Earth now becomes important as we assess the likelihood of the phosphorylation reactions that might follow. By themselves, Ca^2+^ and phosphate precipitate as apatite, and much prebiotic effort has been directed towards making the phosphate in apatite accessible to prebiotic chemistry [51]. However, in a system containing Mg^2+^, sulfate, and borate, the ions partition differently (Figure 9). Here, Mg^2+^ and borate capture the phosphate as the magnesium borophosphate mineral lüneburgite, leaving behind the Ca^2+^ and SO_4_^2−^ to form gypsum. This segregation is not only seen in the laboratory, but also in the field, with numerous reports of co-occurrence of gypsum and lüneburgite [52]. Three specimens of natural lüneburgite, provided by Renato Pagano and Robert Lavinsky, are shown in Figure 9.

**Figure 10:**
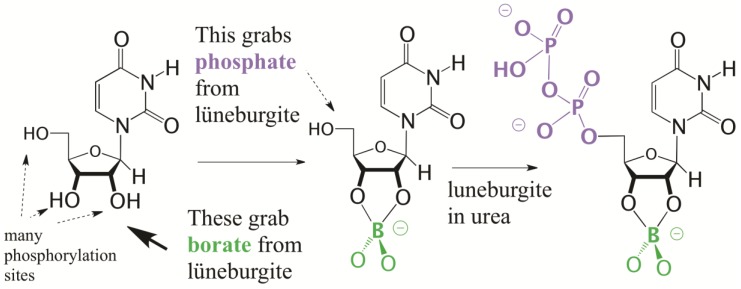
Lüneburgite selectively phosphorylates the 5′-position of nucleosides, since the 2′ and 3′ positions are protected by borate.

Lüneburgite displays an interesting reactivity with nucleosides. The adjacent 2′,3′-diol of the nucleoside captures the borate from lüneburgite, eroding the mineral and freeing its phosphate. If dried in urea under conditions simulating a warm Death Valley, the outcome is 5′-phosphorylation of the nucleoside; the 2′- and 3′-hydroxyl groups of the nucleoside are protected by borate (Figure 10) [53]. The 5′-monophosphate is the principal product; the 5′-diphosphate is formed in detectable amounts, however.

**Figure 11:**
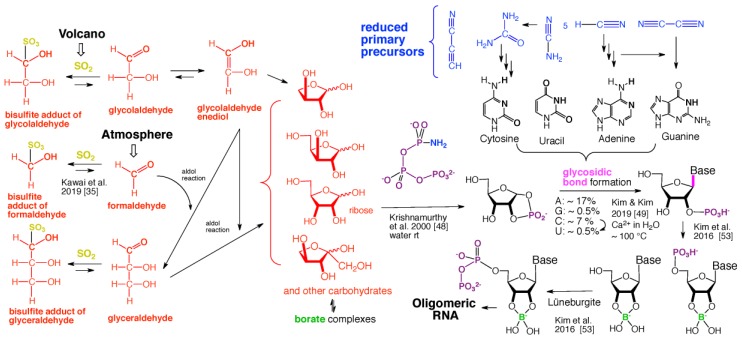
Summary of chemical evolution, all difficult to have avoided in the described Hadean environment, hypothesized to give oligomeric RNA. The silica phases and their relation to oligomeric RNA are described in Reference [55]. Again, stereochemistry is arbitrary. In fact, no hypothesis is offered to generate homochirality among the building blocks presented.

The ability of borate to protect the 2′- and 3′-hydroxyl groups of nucleosides allows cyclic trimetaphosphate to be useful prebiotically in other phosphorylation reactions. The triphosphate is presumed to be a metastable intermediate in the formation of diphosphates. Heating and drying in lipids is an alternative proposed by David Deamer [54] (Figure 11). A variety of silicaceous phases generate oligomeric RNA from these [55], Biondi et al. unpublished].

## 4. The Need for Reduced Precursors

The overall model for this path-hypothesis is captured in Figure 11. Here, an astute reader will recognize a paradox. The introduction of this paper reviewed literature that makes a compelling case for an FMQ mantle delivering correspondingly oxidized minerals to the surface of the Earth beneath a redox-neutral atmosphere. Advantage has been taken of this model throughout this discussion, which has relied on minerals in oxidized forms (borate, phosphate, molybdate, sulfite, and, via disproportionation, sulfate), all accessible to an FMQ mantle and crust. We explicitly excluded formation in a Hadean redox-neutral atmosphere the ammonia, HCN, HCCCN, and other “blue compounds” (Figure 11) that the proposed path-hypothesis now requires.

The likely impact history of Earth, however, provides these compounds in a model that is consistent with quantitative analyses of the amount of siderophilic “iron-loving” metals (e.g., Au, Pt, Ir, Os) in the Earth’s mantle [56]. After core closure, these siderophiles likely were delivered by one (or a few) relatively large impacting bodies; this small number is suggested by the relative amounts of siderophiles on Earth and the Moon. These bodies, in turn, would have had their own iron cores, which would have likely shattered on impact, delivering molten iron to the Hadean atmosphere above an oxidized mantle.

The Fe^0^ delivered by an impactor of this size to the atmosphere could not *not* have reduced water, N_2_, CO_2_, and other gases. The impactor also could not *not* have delivered the Ni^2+^ (about 20% of a typical iron meteorite) needed for nucleoside diphosphate formation (Figure 11). It could not *not* have delivered NH_3_ needed to activate cyclic trimetaphosphate (or a reactivity equivalent) to generate ribose-1,2-cyclic phosphate (Figure 11). And, of course, it could not *not* have allowed atmospheric formation of HCN, HCCCCN, NCCN, H_2_NCN, and other well recognized precursors of nucleobases, as well as their hydrolysis products, including formamide, urea, and cyanoacetaldehyde. Only the amounts and ratios of these reduced species depend on the detailed composition of the impactor and the resulting atmosphere.

The model is agnostic with respect to the size and the date of the relevant impactor. All that is important is that the impact that enables RNA formation not be followed by a subsequent impact that is planet-sterilizing. Thus, the most probable time for the path-hypothesis to operate is soon after the last sterilizing impact, as this impact is likely to have delivered the most and most persistent reduced species to the surface. These are needed not only to create the first Darwinian RNA systems, but also to have fed those systems during any period where they were heterotrophic.

For example, the Mars-sized body (10^25^ kg, Theia) proposed to have caused formation of the Moon is too large; it re-opens the core. Moneta, which is proposed to be ~10^23^ kg (about the size of our Moon) to be able to deliver the late veneer of siderophiles [57], was too small to re-open the core, but nevertheless would have created lava oceans on the surface and re-set available geological clocks; it was certainly sterilizing. A 10^21^ kg Ceres-sized impactor is closer to the boundary between sterilizing and non- sterilizing impactors; it would have created a reducing atmosphere of shorter duration, without resetting the clocks that we find on Earth. A still smaller 10^20^ kg Vesta-sized impactor would neither have sterilized the Earth nor reset the clocks, but still would have transiently generated a productively reducing atmosphere.

Here, however, the important word is “transiently”. An atmosphere that gets its reducing power from the dispersion in the atmosphere of molten iron from an impactor does not keep that reducing power for long. Gases continue to come from the mantle, which is not reduced by any impactor smaller than the hypothetical Moneta, and not much reduced even by Moneta itself. Further, as noted above, the reducing feature of the atmosphere is lost as H_2_ gravitationally escapes to space. With Moneta, which is modeled to have generated as much as 90 bars of H_2_ [58,59], the half-life for the restoration of an unproductive redox neutral atmosphere is tens of millions of years. For a Vesta-sized impactor, the half-life for the loss of a productive atmosphere is much shorter, measured in the tens of thousands of years.

For those hoping to accumulate RNA precursors over geologic periods of time, the bigger the impactor the better. However, most modern models for the impact history of the Earth have impactor size monotonically decreasing over time. Thus, a Ceres-sized impactor may have occurred 1 ± 1 times after a Moneta-sized impactor [59,60]. If it occurred, it was likely sterilizing, and therefore it was most likely to be the impactor most relevant to this hypothesis. In contrast, if it did not occur, and Moneta was the last sterilizing impactor, Moneta is likely to be the most relevant, because it created the longest-lived productively reducing atmosphere. The later Vesta-sized impactors (of which several were likely) are conceivably important, but RNA formation would need to have occurred rapidly after their impact(s). This is, of course, entirely possible, even if it seems to have lower mass balance probability. However, it should be remembered that a productive atmosphere, in this view, would still be needed to “feed” nascent Darwinism even after it got started during the time when it was heterotrophic.

One final consideration might influence one’s preferences of the timing of events outlined in Figure 11: the amount of semi-arid dry land at the relevant time on Earth. Estimates for the amount of continental crust in the Hadean cover a wide range [60], indicating the challenges of modeling this feature of early Earth. Given issues related to buoyancy, the amount of continental crust need not translate directly into the amount of dry land. Of course, a very large impactor would have reduced much of the water, increasing the amount of dry land until a redox neutral atmosphere returned.

Overall, as one referee pointed out, a summary must show when each of the substrates required for the path-hypothesis was present, and in what concentrations, in what combinations, and for what length of time. This is captured in Table 1.

## 5. Conclusions

We present here a relatively direct route to give RNA triphosphate building blocks from a Hadean FMQ mantle, SO_2_-containing volcanic gases, and well-accepted processes that created substantial amounts of HCHO and catalytic amounts of glycolaldehyde in the Hadean atmosphere. In chemistry that could not *not* have happened, these would have generated stable bisulfite addition products that would have rained to the surface. There, in a constrained aquifer, they must have slowly released reactive species, and thus generated higher carbohydrates via aldol reactions. Here, neither surface-level photochemistry nor materials requiring a reducing atmosphere for their formation are required. Further, this chemistry would have been difficult to prevent.

The formation of higher carbohydrates is self-limited by bisulfite in this process. As sulfonates dissociate to give reactive C=O bonds in a constrained aquifer, bisulfite accumulates and captures with increasing efficiency higher C=O carbohydrate products. Further, if present, borate minerals would have controlled the aldol reactions that would have occurred on any semi-arid environments that captured precipitating sulfonates. All of these processes have kinetically studied laboratory correlates.

Further, warm semi-arid land with phosphate could not *not* have had phosphate anhydrides that, with ammonia, yield carbohydrate cyclic phosphate derivatives that react with nucleobases to directly form the canonical nucleosides. These nucleosides are phosphorylated by magnesium borophosphate minerals and/or trimetaphosphate with Ni^2+^-borate to give nucleoside 5′-di and 5′-triphosphates, which can oligomerize to RNA via a variety of mechanisms on silica supports.

The reduced components required for this process came from mid-sized (10^23^–10^20^ kg) impacts that almost certainly occurred during the Hadean. Their metallic iron-nickel content could not have helped but to have generated the ammonia, HCN, nucleobases, and other reduced species in the Hadean atmosphere during the transient period of time when it was out of redox equilibrium with the mantle. Anything of Vesta size or larger could not *not* have created a usefully productive reducing atmosphere, whose persistence would have been longer with larger impactors.

Many of these processes also have kinetically studied laboratory correlates. Missing are the quantitative details that would allow us to assess the relative proportions of carbohydrates made, for example, the ratios of four-carbon carbohydrates (e.g., threose and erythrose), branched five-carbon carbohydrates, and linear five-carbon ketose and aldose carbohydrates, the last represented by ribose and arabinose. These details aside, these compounds could not *not* have formed on a Hadean subaerial surface intermittently associated with water.

Also missing from this path-hypothesis is homochirality. This path-hypothesis offers no mechanism to get nucleosides that all have the same enantiomeric form. Because the Simons Collaboration had team members addressing this issue, the Templeton Foundation consortium focused elsewhere. We are looking forward to the Simons Collaboration offering candidate solutions to this very important chirality problem.

## Figures and Tables

**Table 1:** Summary of when, how much, and how long.

Compound	From Where?	When Began	How Much	How Long
SO_2_	Volcanic from mantle	When FMQ mantle established	~10^9^ kg/year	Continuous
CO_2_	Volcanic from mantle	Ditto	Atmospheric	Continuous
H_2_O	From mantle	Ditto		Continuous
N_2_	From mantle	Ditto	Atmospheric	Continuous
HCHO	Photochemistry of CO_2_ and H_2_O upper atmosphere	Continuous until impact; returning after	~10^9^ kg/year	Continuous
HOCH_2_CHO	Photochemistry of CO_2_ and H_2_O upper atmosphere; electrical discharge	Continuous	~10^3^ kg/year	Continuous
Bisulfite C=O addition products	Combination of atmospheric SO_2_ + C=O compounds	Continuous	~3 mg/m^2^/yr	Continuous
Borate minerals	Basalts from B-enriched melts; tectonics not needed (see Mars)	When FMQ mantle established	Depending on concentration in hydrosphere	Continuously present
Phosphate minerals; note also phosphite availability	From igneous apatites, etc.	When FMQ mantle established		Continuously present; availability dependent on pH
Fe^0^	From last sterilizing impactor	Between 4.7 and 4.2 Ga	Depending on impactor size	Depending on impactor size; 10^3^ years.
HCCCH, HCN, NCNH_2_, NH_3_	Reduction in atmosphere by Fe^0^ from last sterilizing impactor of N_2_, H_2_O, CO_2_	Between 4.7 and 4.2 Ga	For example, HCCCN is as much as 9% of total yield	Depending on impactor size, produced for 10^4^–10^7^ years.
Adenine, uracil, guanine, cytosine	Produced surface from HCN, NH_3_, HCCCH, NCNH_2_,	Between 4.7 and 4.2 Ga	Estimated 20 mg/m^2^/year	Depending on impactor size, produced for 10^4^–10^7^ years.
